# Role and mechanism of DNA methylation and its inhibitors in hepatic fibrosis

**DOI:** 10.3389/fgene.2023.1124330

**Published:** 2023-03-28

**Authors:** Shi-Yi Lyu, Wang Xiao, Guang-Zu Cui, Cheng Yu, Huan Liu, Min Lyu, Qian-Ya Kuang, En-Hua Xiao, Yong-Heng Luo

**Affiliations:** ^1^ Department of Radiology, The Second Xiangya Hospital, Central-South University, Changsha, Hunan, China; ^2^ Department of Gastrointestinal Surgery, The Second Xiangya Hospital, Central-South University, Changsha, Hunan, China; ^3^ XiangYa School of Medicine, Central South University, Changsha, Hunan, China

**Keywords:** hepatic fibrosis, DNA methylation, epigenetics, hepatic stellate cells (HSCs), DNA methylation inhibitors

## Abstract

Liver fibrosis is a repair response to injury caused by various chronic stimuli that continually act on the liver. Among them, the activation of hepatic stellate cells (HSCs) and their transformation into a myofibroblast phenotype is a key event leading to liver fibrosis, however the mechanism has not yet been elucidated. The molecular basis of HSC activation involves changes in the regulation of gene expression without changes in the genome sequence, namely, *via* epigenetic regulation. DNA methylation is a key focus of epigenetic research, as it affects the expression of fibrosis-related, metabolism-related, and tumor suppressor genes. Increasing studies have shown that DNA methylation is closely related to several physiological and pathological processes including HSC activation and liver fibrosis. This review aimed to discuss the mechanism of DNA methylation in the pathogenesis of liver fibrosis, explore DNA methylation inhibitors as potential therapies for liver fibrosis, and provide new insights on the prevention and clinical treatment of liver fibrosis.

## 1 Introduction

Liver fibrosis is caused by long-term liver damage and excessive deposition of connective tissue mediated by myofibroblasts. Chronic liver fibrosis develops into liver cirrhosis, and up to 30% of liver cirrhosis cases eventually progress into hepatocellular carcinoma (Zhang et al., 2019). Hepatic stellate cells (HSCs) become activated, transform into myofibroblasts, and secrete type I collagen (COL1A1). High levels of extracellular matrix (ECM) and various hepatic fibrosis-promoting factors are central components of fibrosis (Wu et al., 2021). Therefore, inhibiting HSC activation and proliferation as a therapeutic target to reduce ECM deposition is an important method for treating liver fibrosis. Epigenetic changes involve alterations in the gene sequence without heritable changes in the gene function. DNA methylation is an important mode of epigenetic regulation that is closely related to liver fibrosis (Shen and Luo, 2020). Methylation is a direct chemical modification of DNA, which occurs on the fifth carbon atom of the cytosine nucleotide ring to form 5-methylcytosine (5 mC), most commonly found in cytosine-phosphate-guanine (CpG) dinucleotides (Chen and Zhang, 2020). DNA methylation protects the corresponding DNA from specific restriction enzymes and regulates gene expression (Duncan et al., 2014). Regulation of DNA methylation is a potential therapeutic target for fibrosis (Shen and Luo, 2020). This review discusses the DNA methylation mechanism involved in the occurrence and development of hepatic fibrosis and research progress on methylation inhibitors in the treatment of hepatic fibrosis.

## 2 Pathogenesis of hepatic fibrosis

### 2.1 Liver fibrosis process

Hepatic fibrosis is a highly integrated dynamic process of molecules, cells, and tissues, that aims to limit liver damage in chronic liver diseases (such as alcohol abuse and viral infection), but leads to the gradual accumulation of ECM (Kong et al., 2020). The pathogenesis of liver fibrosis is primarily associated with the following three factors. First, several inflammatory factors, including platelet-derived growth factor, transforming growth factor-beta (TGF-β), and some chemokines, are released after liver injury, activating cell proliferation, migration, and ECM secretion, and promoting the transdifferentiation of mesenchymal cells into myofibroblasts. Second, in the healthy liver, the synthesis and degradation of ECM are precisely regulated, and thus the liver content is in dynamic equilibrium. In contrast, in chronic liver injury, HSCs synthesize a large amount of ECM and secrete matrix metalloproteinase (MMP) inhibitors [including tissue inhibitors of metalloproteinases (TIMPs)] to interfere with ECM degradation, resulting in homeostasis imbalance, ECM remodeling, and liver fibrosis (Ren et al., 2019). Lastly, HSCs and Kupffer cells settled in the liver or fibroblasts, pericytes, and epithelial cells derived from the bone marrow are transdifferentiated into myofibroblasts to repair the injured liver (Xu et al., 2014), in which HSC activation is the main source of myofibroblasts.

### 2.2 HSC activation and liver fibrosis

HSCs not only coordinate ECM deposition in the injured liver, but also are the main effectors of liver inflammation (Gao et al., 2020). HSCs are static in the physiological state and located in the perisinusoidal space. HSCs metabolize and store 80% of the body’s vitamin A, MMPs, and their inhibitors TIMPs, and express cytokines and receptors, which play an important role in vitamin A metabolism, regulation of ECM homeostasis and remodeling, and regulation of hepatocyte regeneration (Ren et al., 2019). In the process of liver fibrosis, HSCs transdifferentiate into proliferative, migratory, and contractile myofibroblasts referred to as activated HSCs, that exhibit the secretory characteristics of fibrotic cells (Tsuchida and Friedman, 2017). Various pro-fibrogenic factors released after liver injury, such as TGF-β, interleukin (IL)-6, IL-17, and connective tissue factors, directly stimulate the activation and proliferation of HSCs, promoting their migration to the injured site, secretion of different matrix components, and excessive deposition of ECM. This results in damage to liver function, morphological changes in the liver parenchyma, and formation of fibrous cords or scars, thus generating liver fibrosis. However, hepatic fibrosis is reversible, and the deposition of ECM can be regulated. The regression of hepatic fibrosis is often accompanied by apoptosis, aging, or inactivation of HSCs and degradation of the ECM (Zhao and Chen, 2019). Activated HSCs are factors in the pathogenesis of hepatic fibrosis, suggesting that HSCs may be used as therapeutic targets to interfere with the intracellular signal pathway when the root cause of a recurrent or chronic injury is unknown. Such molecular biology techniques may include reversing or blocking DNA hypermethylation to inhibit HSC activation and proliferation, promoting HSC apoptosis, aging, or inactivation, and promoting the degradation of ECM to reverse liver fibrosis.

### 2.3 DNA methylation and liver fibrosis

**FIGURE 1:**
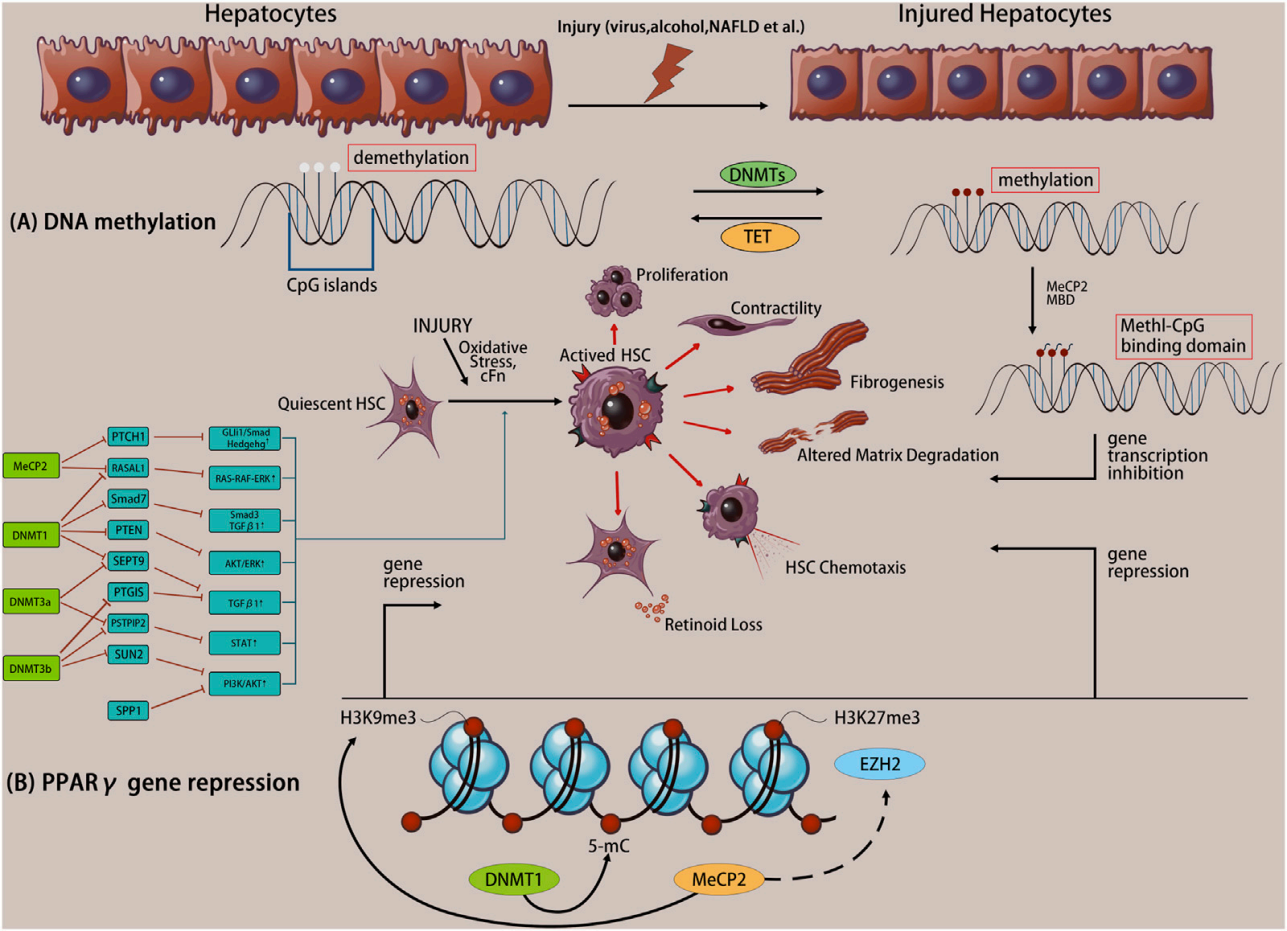
Liver injury initiates the transdifferentiation of quiescent hepatic stellate cells (HSCs) to their activated phenotype. Altered DNA methylation causes specific gene expression/repression processes in response to specific phenotypic changes, including proliferation, contractility, fibrosis, matrix degradation, chemotaxis, retinoid loss, etc. **(A)** The molecule mechanism of DNA methylation. **(B)** Example of coordinated DNA methylation regulation of PPARγ transcriptional repression in activated HSCs.

DNA methylation *via* catalysis by DNA methyltransferase (DNMT) is mediated by a selective covalent bond between a CpG island 5-cytosine and the methyl group provided by S-adenosylmethionine to form 5 mC (Kong et al., 2014). DNA methylation is regulated by two mechanisms. Firstly, DNA that is unmethylated on both strands is methylated, called *de novo* methylation, which prevents transcription factors from binding to genes, resulting in transcriptional repression. Secondly, one of the strands of DNA in a double strand has been methylated and the other unmethylated strand is methylated, called maintenance methylation. Methylated DNA is recognized by a protein called the methyl-CpG-binding domain (MBD), which recruits other proteins to sites, such as histone deacetylases and other chromatin remodeling factors that can modify histones, to form dense, inactive heterochromatin, thereby inhibiting gene transcription. Gene promoters contain one or more clusters of CpG islands, and most promoters are in the non-methylated state, while the gene body is usually in the methylated state (Ooi et al., 2007) (Figure 1A). Notably, although DNA methylation of CpG islands is clearly associated with transcriptional repression, the function of DNA methylation in CG-deficient promoters remains unclear. Typically, promoter hypermethylation silences gene expression, whereas hypomethylation upregulates gene expression, and overall promoter hypermethylation is negatively correlated with gene expression (Liang Y. et al., 2013). In contrast, gene body hypermethylation is positively associated with active transcription, which regulates splicing and represses the activity of transcription units (codons, promoters, or transposons) within genes, and the function of gene body methylation is currently unknown (Shen and Laird, 2013; Sun et al., 2018).

There are five members of the DNMT family, including three active enzymes, including DNMT1 which is responsible for maintaining the DNA methylation status during DNA replication, and DNMT3a and DNMT3b which mediate *de novo* methylation of CpG islands to generate new epigenetic marks (Han et al., 2020). During the differentiation of HSCs into fibroblasts, approximately 400 DNA sites (including DNMT3a and DNMT3b) undergo methylation, resulting in over 20% DNA methylation (including hypomethylation and hypermethylation), which is related to gene transcriptional activation or repression.

Methyl-CpG-binding protein 2 (MeCP2) is a transcriptional repressor that specifically binds to methyl-CpGs and represses transcription from methylated gene promoters, or is a complex member of DNMT1 involved in DNA methylation maintenance (Lao et al., 2010). MeCP2 binds to the methyl-CpG sequence in the promoter region of peroxisome proliferator-activated receptor gamma (PPARγ), regulates the repressive H3K9me3 modification enzymes, and inhibits PPARγ transcription initiation and transcription of the PPARγ downstream coding region. Furthermore, MeCP2 promotes enhancer of zeste homolog 2 expression, activates HSCs, and increases collagen secretion *in vitro* and *in vivo* (Tahiliani et al., 2009) (Figure 1B). The PPARγ gene is involved in the regulation of adipogenesis, maintenance of the quiescent state in HSCs, and the non-fibrotic phenotype (Huang et al., 2019). Thus, PPARγ gene silencing is necessary for HSC activation. Conversely, MeCP2 also acts as a transcriptional activator (Mellen et al., 2012). MeCP2 actively regulates the expression of absent small or homeotic 1 (ASH1) during HSC transdifferentiation and promotes the transcription of classic profibrotic genes (including COL1A1 and TGF-β1) (Perugorria et al., 2012), coupled with the upregulation of multi-fiber genes (Mou et al., 2022), which ultimately aggravates fibrosis. Additionally, HSCs isolated from MeCP2-knockout mice display decreased expression of myofibroblast marker genes (such as α-smooth muscle actin [SMA] and COL1A1) compared with that in controls, and MeCP2-knockout mice are protected from carbon tetrachloride (CCl_4_)-induced liver fibrosis (Mann et al., 2010). MeCP2 is involved in the maintenance of DNA methylation, and silencing or knocking down the MeCP2 gene promotes PPARγ transcription, inhibits HSC activation, and alleviates the progression of liver fibrosis. Therefore, MeCP2 and DNA methylation may provide the molecular mechanism for permanent activation and fibrosis of fibroblasts in the liver.

## 3 Liver fibrosis-related genes and DNA methylation

### 3.1 Smad gene

Smad proteins are a class of transcriptional coordinators that must interact with other DNA-binding proteins to regulate the transcription of target genes. The Smad family of proteins is divided into three categories: receptor-activated or pathway-restricted Smad (R-Smad), common pathway-restricted Smad (co-Smad), and inhibitory Smad (I-Smad). Smad2/Smad3, a downstream factor of Smad signaling, is considered a key regulator of TGF-β signaling in tissue fibrogenesis (Yao et al., 2012), and more importantly, Smad7 is a potential major transcriptional repressor of HSC activation and liver fibrosis *in vitro* and *in vivo*, regulating Smad2/Smad3 phosphorylation (Xu et al., 2016). Firstly, TGF-β promotes hepatic fibrosis by inducing HSCs synthesis of TIMPs to inhibit ECM degradation. Secondly, TGF-β promotes fibrosis progression by activating downstream activating mediators, namely, Smad2/Smad3 phosphorylation, which translocate Smad2/Smad3 to the nucleus and then bind to pro-fibrotic DNA sequences that depend on Smad3 to function, promoting the expression of fibrotic proteins, such as COL1A1, α-SMA and epithelial calmodulin E-cadherin (Masszi and Kapus, 2011). At the same time, phosphorylated Smad2/Smad3 positively feeds back into the TGF-β pathway, exacerbating the development of liver fibrosis. This process is negatively regulated by the inhibitory Smad7 (Peng et al., 2022). Bian et al. (2014) reported that silencing DNMT1 increased Smad7 expression and reduced Smad2/Smad3 phosphorylation, significantly reduces TGF-β1-induced HSC activation. DNA methylation is involved in the reduction of Smad7 expression during HSC activation. Besides, overexpression of Smad7 in rats with common bile duct ligation-induced hepatic fibrosis completely blocked TGF-β signaling and protected against liver injury, as evidenced by inhibition of Smad2/Smad3 phosphorylation, blocking HSC activation and leading to reduced COL1A1 expression. Moreover, Smad7 also abrogated the TGF-β-dependent proliferation of HSCs. Smad7 promotes HSC activation and liver fibrosis through epigenetic suppression of DNA methylation/low expression. smad7 is protective and Smad3 is pathogenic. Although α-SMA expression was not reduced by Smad7, the actin cytoskeleton’s fibrillar structure was destroyed when stained with anti-alpha-SMA antibodies (Dooley et al., 2003; Dooley et al., 2008). These results may help us comprehend the role of Smad7 inactivation in liver fibrosis and open the path for further research into its potential targets in the development of treatment approaches.

### 3.2 Phosphatase and tensin homologgue (PTEN)

PTEN is a tumor suppressor gene that negatively regulates proliferation, survival, cell cycle, adherent spots and cell migration, mainly through PI3K-dependent and independent mechanisms. PTEN can also exert inhibitory functions through its protein phosphatase activity. In addition to influencing tumorigenesis, epigenetic processes play a key role in fibrogenesis and fibrosis. When TGF-β1 secretion is increased in the liver, the ERK pathway is activated, which promotes HSC activation and ECM secretion (Li et al., 2013). PTEN inhibits the ERK signal transduction pathway *via* protein tyrosine phosphatase activity and inhibits fibrosis. Moreover, the downregulation or loss-of-function of the PTEN gene leads to the accumulation of phosphatidylinositol-3,4,5-triphosphate, subsequently enhancing phosphatidylinositol-3-kinase (PI3K)/protein kinase B (AKT) and ERK activity, increasing mitogen signaling and/or decreasing apoptosis, and ultimately leading to liver fibrosis (Yang et al., 2015). PTEN promoter hypermethylation/low expression or deletion has been observed in TGFβ-induced activated HSC-T6 cells *in vitro* as well as in a CCl_4_-induced mouse hepatic fibrosis model (Guan et al., 2022), which is mediated by DNMT1 (Zhu et al., 2020; Song H. et al. 2021; Guan et al., 2022). Therefore, targeting PTEN or DNMT, *via* silencing the DNMT1 gene or using DNMT inhibitors to prevent PTEN hypermethylation and expression deficiency, and negatively regulate the AKT/ERK pathway, thereby inhibiting HSC activation and alleviating liver fibrosis.

### 3.3 Patched-1 (PTCH1)

PTCH1 protein is a component of the hedgehog (Hh) signaling pathway (Bragelmann et al., 2021). The Hh signaling pathway is closely related to tissue homeostasis, cell proliferation, differentiation, apoptosis, and migration, and plays a key role in cell growth, differentiation, and animal embryonic development. Furthermore, the Hh signaling pathway is associated with the activation of HSCs. When the Hh protein is absent, PTCH1 binds to its ligands, and activated PTCH1 inhibits smoothened (Smo), thereby reducing Smo activity and inhibiting the downstream signaling pathways. When PTCH1 binds to the Hh protein, PTCH1 is inhibited, which relieves the inhibitory effect of PTCH1 on Smo and activates Smo. This in turn activates the transcription factors Gli1 and Smad3 and expression of Ptch, Wnt, epidermal growth factor, and other target genes, and promotes the secretion of TGF-β1, thus activating HSCs and increasing secretion of COL1A1 and α-SMA (Yang et al., 2013). PTCH1 expression is related to its promoter methylation level, which affects Hh signaling pathway expression. Firstly, *in vitro* activation of HSCs and CCl_4_-induced liver fibrosis models in mice showed that PTCH1 affects HSC activation through the Gli1 and Smad3 signaling pathways. When PTCH1 is hypermethylated, PTCH1 expression is downregulated and HSCs are activated (Yang et al., 2022), while ALKBH5 upregulates PTCH1 expression and improves or alleviates liver fibrosis (Chen et al., 2012). Second, deletion of ALKBH5 was reported to be associated with worsening clinical liver fibrosis in collected clinical liver fibrosis samples. It was confirmed that ALKBH5 expression was significantly lower in human liver fibrosis tissues in mRNA, protein, and immunohistochemical staining compared to controls, and that increased expression of COL1A1 and α-SMA was also consistent with the severity of liver fibrosis detected with Sirius red and Masson trichrome staining (Yang et al., 2022). And hepatic myofibroblasts with MeCP2 siRNA knockdown expressed more PTCH1 mRNA and protein (Yang et al., 2013). These data suggest that DNA methylation and MeCP2 may provide the molecular mechanism for silencing PTCH1. Hh signaling pathway and PTCH1 gene hypermethylation are closely associated with cell proliferation and liver fibrosis, and these findings may be applied to the diagnosis and treatment of patients with liver fibrosis.

### 3.4 RAS protein activator like 1 (RASAL1)

The Ras gene is a proto-oncogene that is present in several human cells. The encoded Ras protein is involved in cell proliferation, differentiation, and cytoskeleton construction. In healthy cells, the RAS protein is in an inactive state. When the RAS protein binds to GTP in an activated state, the downstream Ras-Raf-MEK-ERK signaling pathway is abnormally activated, and excessive proliferation signals are transmitted, leading to uncontrolled cell proliferation and the development of tumors (Chen K. et al., 2021). The RASAL1 gene is a tumor suppressor gene that encodes the RASAL1 protein, a member of the Ras-GAP family with GTPase activity and converts active RAS-GTP to inactive RAS-GDP, regulating normal cell growth, differentiation and proliferation (An et al., 2020). There is evidence that hypermethylation is the main mechanism for downregulating RASAL1 gene expression and is associated with fibrotic diseases (Mann et al., 2010). and that epigenetic RASAL1 silencing leads to fibroblast activation and fibrogenesis, similar to the ability of cancer cells to proliferate in a growth factor-independent manner following RASAL1 silencing (Kolfschoten et al., 2005; Jin et al., 2007). Moreover, siRNA knockdown of the MeCP2 gene in HSC-T6 cells inhibits the Ras signaling pathway, resulting in upregulation of RASAL1 mRNA and protein expression in myofibroblasts and suppression of HSC activation and proliferation (Mann et al., 2010). It was demonstrated that the promoter of the methylated RASAL1 gene binds to MeCP2 to repress RASAL1 transcriptional expression and maintain an active RAS-GTP state. According to Bechtel’s research, the low expression of RASAL1 in mice renal fibrogenesis was caused by hypermethylation of the RASAL1 promoter, which is mediated by DNMT1 (Bechtel et al., 2010). Tao et al. (2011) demonstrated that the methylation inhibitor DAC reversed the loss of RASAL1 expression during HSCs proliferation, increasing RASAL1 expression and inhibiting α-SMA expression. This suggests that the hypermethylated RASAL1 gene plays a role in perpetuating fibroblast activation through the imprinting pathway of fibroblast activation and ultimately fibrogenesis.

### 3.5 Prostacyclin synthase (PTGIS)

PTGIS is a member of the CYP8 family that catalyzes the conversion of prostaglandin H2 to prostacyclin I2 (PGI2) (Yu et al., 2017). Both PTGIS and PGI2 exert inhibitory effects on tumor proliferation (Khatun and Ray, 2019; An et al., 2020). PTGIS gene methylation is mediated by the methyltransferases DNMT1 and DNMT3b. The expression of PTGIS is decreased in colon cancer, but increased after the application of the methylation inhibitor DAC, indicating that methylation of the PTGIS gene contributes to the reduction in PTGIS gene expression (Cheng et al., 2021). Pan et al. (2018) found that the promoter of the PTGIS gene was detected to be hypermethylated and under-expressed in TGFβ1-activated HSC-T6 and a CCl_4_-induced liver fibrosis mouse model, whereas enforced expression of PTGIS *in vitro* and *in vivo* could counteract the activation of HSCs and even cause apoptosis, thereby alleviating liver fibrosis. This has been confirmed by detecting reduced protein and mRNA expression levels of COL1A1 and α-SMA and downregulation of serum ALT/AST levels in activated HSC-T6 cells and primary HSCs isolated from a liver fibrosis mouse model. DNA methylation of PTGIS plays an important role in the progression of liver fibrosis and activation of HSCs, implying that targeting PTGIS has a therapeutic potential ability to treat liver fibrosis.

Notably, PTGIS expression is elevated in the early stages of CCl_4_-induced liver fibrosis mice models only to be downregulated at a later stage (Pan et al., 2018). Cebola et al. (2015) found that PTGIS misregulation leads to the accumulation of pro-inflammatory signals. Furthermore, most types of liver injury damage epithelial cells, which leads to the release of inflammatory cytokines such as TGF-β1, IL-6, and TNF (Pellicoro et al., 2014), followed by activation of HSCs by TGF-β1. Therefore, elevated PTGIS expression in the early stages may be attributed to its positive feedback on the inflammatory response. It suggests that interventions for inflammation in addition to methylation aspects are important in the treatment of liver fibrosis.

### 3.6 Sad1/UNC84 domain-containing protein 2 (SUN2)

SUN2, is an inner nuclear membrane protein with an evolutionarily conserved Sad1/UNC-84 homology (SUN) domain at the C-terminus of the protein (Turgay et al., 2010) and is a key component of the connection between the nuclear skeleton and cytoskeleton complex, highly conserved in all eukaryotes and widely expressed in various organs and tissues (Hsieh et al., 2014). SUN2 is also a novel anticancer drug candidate with the ability to inhibit the proliferation and migration of cancer cells and promote their apoptosis. Chen et al. (2018) found that enforced expression of SUN2 inhibited the activation of HSCs and exerted antifibrotic effects in TGF-β1-activated HSC-T6 cells. And in CCl_4_-induced liver fibrosis mice, DNMT3b mediates hypermethylation of the SUN2 gene at CpG sites, leading to a reduction in SUN2 expression, induction of HSC activation, and liver fibrosis. Additionally, Lei et al. (2012) found that overexpression of SUN2 alleviates excessive DNA damage in SUN1/2-knockout mouse embryonic fibroblasts. Moreover, SUN2 is necessary for maintaining genomic stability, and DNA damage plays an important pathogenic role in liver fibrosis. SUN2 deficiency induces DNA damage and HSC activation, leading to liver fibrosis. Therefore, hypermethylation of the SUN2 gene mediates the decrease in SUN2 expression in liver fibrosis. Inhibiting DNMT3b and upregulating SUN2 expression may maintain gene stability and reduce the damage caused by fibrosis.

### 3.7 Proline-serine-threonine-phosphatase-interacting protein 2 (PSTPIP2)

The PSTPIP2 gene is located on chromosome 18 and belongs to the Pombe Cdc15 homology protein family (Yang Y. et al., 2018). PSTPIP2 influences cell proliferation, apoptosis, and inflammatory cytokine secretion, and plays an important role in tumor autoimmunity and other diseases (Chitu et al., 2012). In the progression of liver fibrosis, epigenetic mechanisms are key factors that control the phenotype of macrophages. Hypermethylation of PSTPIP2 is mediated by the methyltransferases DNMT3a and DNMT3b in LPS-induced RAW264.7 cells (Yang Y. et al., 2018). By secreting cytokines and chemokines that stimulate HSCs, activated hepatic macrophages promote liver fibrosis (Friedman, 2008; Cohen-Naftaly and Friedman, 2011). The expression of PSTPIP2 is significantly decreased after partial methylation of PSTPIP2. PSTPIP2 gene overexpression inhibits the expression of classical macrophage (M1) markers by inhibiting signal transducer and activator of transcription 1 (STAT1) activity. Furthermore, PSTPIP2 overexpression promotes STAT6 activity which enhances the expression of alternatively activated macrophage (M2) markers, decreases M1/M2 expression. And PSTPIP2 is enriched in M2-type macrophages, which produce many anti-fibrotic factors, such as IL-10, much more than the small number of fibrotic factors, such as TGF-β and IL-13, that are produced, reducing the inflammatory response and improving liver fibrosis. In mouse liver macrophages isolated from a CCl_4_-induced mouse liver fibrosis model, the expression of PSTPIP2 is significantly decreased after partial methylation of PSTPIP2(57). Xu et al. (2022) reported that DNMT3a, a major regulator of PSTPIP2 expression, directly binds to the PSTPIP2 promoter, resulting in PSTPIP2 hypermethylation and low expression, and that silencing DNMT3a significantly restored ethanol-induced low expression in mouse hepatic macrophages. Furthermore, PSTPIP2 may be a potentially critical factor in regulating macrophage polarization in hepatic fibrosis. Thus, inhibition of PSTPIP2 methylation provides a promising approach for therapeutic interventions to prevent or treat liver fibrosis, liver inflammation and related diseases.

### 3.8 Septin 9 (SEPT9)

The human septin family contains 14 genes (SEPT1-SEPT14), encoding dozens of different septin proteins. Among them, SEPT9 encodes a GTP-binding protein, which plays a role in cytokinesis, intracellular material transport, cell cycle regulation, and apoptosis (Ivanov et al., 2021). Wu et al. (2017) found that SEPT9 is hypermethylated in TGF-β1-treated mouse HSCs and CCl_4_-treated mouse liver, and SEPT9 protein expression is significantly reduced, leading to increased ECM accumulation and aggravated fibrosis compared with that in controls. This process can be antagonized by methylation inhibitors silencing DNMT3a and SEPT9 overexpression, thereby reducing liver fibrosis, including upregulation of apoptosis-related proteins in myofibroblasts, and reduction of TGF-β1-induced expression of α-SMA and COL1A1. Li et al. (2022) demonstrated that DNMT1 can also mediate methylation of SEPT9. These results say that SEPT9 methylation is a molecular mechanism of liver fibrosis and is significantly correlated with early diagnosis and prognosis of the disease (Xie et al., 2022; Zhao et al., 2022). Whether the methylation of SEPT9 gene in peripheral blood can also be used for the diagnosis and treatment of liver fibrosis in the future needs to be further investigated.

### 3.9 Secreted phosphoprotein 1 (Spp 1)

Spp 1, also referred to as osteopontin, is a ubiquitously expressed secreted phosphoglycoprotein isolated from the mineralized bone matrix and produced by microvascular endothelial cells, fibroblasts, inflammatory cells, and muscle cells (De Luca et al., 2003). SPP1 is a potential oncogenic factor, and is found in tissues, body fluids, and cells under physiological conditions (Stemberger et al., 2014). Silencing of SPP1 inhibits the PI3K/AKT signaling pathway, negatively regulating the ability of various signaling pathways to control cell proliferation, migration, and metabolism (Zhu and Su, 2021). Spp1 expression is increased during many tissue injury repair processes. For example, a reduction in collagen fibers, matrix degradation, and failure of incision healing are observed in a skin incision model in Spp1-knockout mice, suggesting a role for Spp1 in wound repair processes (Call et al., 2008). In Protein-Protein Interaction (PPI) network construction and core gene screening, the modules that include CTGF, TIMP1, SPP1, COL3A1, and other genes are located in the core of the network, which is mainly involved in ECM synthesis, and fibroblast proliferation and transdifferentiation (Heinemeier et al., 2012; Kovacic et al., 2012). Komatsu et al. (2012) analyzed DNA methylation in the liver tissue of rats with CCl_4_-induced liver fibrosis and demonstrated that hypomethylation/high expression of the Spp1 gene activates the PI3K/AKT pathway, inducing HSC activation and ECM secretion. The methylation level of the Spp1 gene promoter region is negatively correlated with its expression. Unlike most well-known genes, the hypomethylation/high expression of the SPP1 gene is closely related to the progression of liver cancer and liver fibrosis (Chen et al., 2019; Song Z. et al., 2021), while in the known liver fibrosis-related genes we described all undergo hypermethylation/low expression, promoting fibrosis development. These findings suggest that hypomethylation of the Spp1 gene and increased secretion of COL1A1 and α-SMA contribute to the development of liver fibrosis. Whether hypermethylation of SPP1 gene can treat liver fibrosis needs to be further investigated.

## 4 DNA methylation inhibitors

DNA methylation inhibitors are mainly divided into two classes: nucleoside and non-nucleoside analogs (Cheng et al., 2019). Nucleoside analogs include cytidine and S-adenoyl-L-homocysteine derivatives, while non-nucleoside inhibitors primarily include natural products and small molecules that do not contain cytidine (Yang C. Y et al., 2018).

### 4.1 Nucleotide analog DNA methylation inhibitors

#### 4.1.1 5-Azacytidine and 5-aza-deoxycytidine

5-azacytidine (5-Aza-CR, 5AC) and DAC (5-Aza-deoxycytidine, 5-Aza-CdR) are two commonly used nucleoside analog DNA methylation inhibitors. Both have been approved by the United States Food and Drug Administration (FDA) for the treatment of myelodysplastic syndrome (Tang and Liu, 2014). 5AC is a naturally occurring pyrimidine nucleoside cytidine ring analog that is incorporated into the RNA and DNA of eukaryotic and prokaryotic cells (Shi et al., 2022). There are two main pathways for 5AC metabolism. First, 5AC is integrated into DNA after being phosphorylated by reductase and covalently bound to DNMT to competitively inhibit DNMT activity and DNA methylation. Second, 5AC preferentially integrates into RNA and requires conversion into a deoxy form before phosphorylation and integration into DNA as phosphorylation and incorporation into RNA disrupt RNA metabolism (Uddin and Fandy, 2021). Thus, the integration efficiency of 5AC in DNA is much lower than that of DAC. In contrast, DAC does not integrate into RNA. Therefore, DAC demethylates DNA at a concentration of five to ten times lower than that of 5AC (Daher-Reyes et al., 2019).

These two drugs both target the S phase of the cell cycle. Although high doses of these compounds induce cytotoxicity, they differ from traditional chemotherapy drugs because low doses do not directly lead to cell death. And, they reverse the hypermethylation of CpG islands in the promoter region of tumor suppressor gene by upregulating genes that are methylated due to fibrosis, such as the RASAL1 gene (Tao et al., 2011), reverse high methylation of tumor-suppressor gene promoter region CpG islands, cleave fibrosis-related, COL1A1, and α-SMA genes, and inhibit HSC activation in fibroblasts (Wu et al., 2017). This reduces the secretion of ECM and COL1A1, relieves liver fiber damage, and reactivates methylation silencing of tumor suppressor genes (Liang S. et al., 2013).

However, the disadvantages of 5AC and DAC are becoming increasingly apparent. First, both are unstable and toxic in aqueous solutions. Second, deamination by cytosine deaminase in the intestinal mucosa and liver leads to rapid inactivation; DAC has a half-life of 15–25 min and a 90% clearance rate within an hour after clinical administration (Griffiths et al., 2013), resulting in poor oral bioavailability. Third, binding of 5AC and DAC to DNA forms irreversible covalent complexes that may lead to DNA mutations (Nakamura et al., 2013). Lastly, high-dose regimens of these drugs lead to bone marrow suppression, anti-metabolic and DNA damaging effects, and eventually cell apoptosis (Tang and Liu, 2014). Therefore, low-dose regimens are currently used in clinical trials (Han et al., 2021), which limits their anti-fibrosis applications. Due to the low oral bioavailability and high cytotoxic effects of 5AC and DAC, new nucleoside analogs are continuously being developed, including zebularine, guadecitabine (SGI-110), and 6-thioguanine (6-TG), which is the third FDA-approved nucleoside analog for acute myeloid leukemia treatment. Within DNA, 6-TG wraps DNMT1 in a covalent binding manner and subsequently degrades it.

#### 4.1.2 Zebularine

The metabolic activation of zebularine, a second-generation DNA methyltransferase inhibitor, resembles that of 5AC, and zebularine is also incorporated into RNA and DNA. Compared with that of 5AC and DAC, zebularine exhibits the advantages of water solubility, stability, low toxicity, and high oral bioavailability (Zhang et al., 2015). The half maximal inhibitory concentration (IC50) of zebularine is approximately seven-fold higher than that of 5AC (120 μM vs 17.4 μM, respectively) in human bladder cancer T24 cells, with a degradation rate lower than 7% within 48 h (Ben-Kasus et al., 2005) and a half-life of 508 h during physiological conditions (Marquez et al., 2003). Therefore, zebularine exerts greater stability and safety effects than DAC and 5AC.

Zebularine also inhibits cytidine deaminase, which confers resistance to nucleoside analogs (Tang et al., 2021). Zebularine can be incorporated into DNA and form covalent complexes with DNMT to competitively inhibit DNMT activity, demethylate tumor suppressor genes, such as SFRP2, Dkk3, and p16, and inhibit tumor cell proliferation, differentiation, and metastasis. Upregulation of tumor suppressor gene expression in cancer cells induces tumor cell apoptosis in esophageal (Zhang et al., 2021) and liver HepG2 (Ding et al., 2015) cancer cells in a dose- and time-dependent manner. In the treatment of fibrosis, zebularine inhibits TGF-β1-induced differentiation of lens epithelial cells into myofibroblasts, thereby reducing the occurrence of fibrosis (Zhou et al., 2011). Furthermore, zebularine downregulates MeCP2 expression in a time- and dose-dependent manner and reverses TGF-β2-induced α-SMA expression, indicating an inhibitory effect of zebularine on fibrosis (Zhou et al., 2011). Huang et al. (2010) used both a DNA methylation inhibitor (DAC or zebularine) and DNMT enzyme-specific small interfering RNA, both of which exert demethylation effects in fibrotic diseases, and found that the methylation level of the prostaglandin E receptor 2 (PTGER2) gene was downregulated and the mRNA and protein expression levels of E prostanoid 2 were upregulated in fibrotic fibroblasts. These methods also restored the responsiveness of non-fibrotic cells to the lipid mediator prostaglandin PGE2, a protective factor that prevents fibroblasts from displaying fibrotic activity. Lastly, AKT signaling was increased, driving PTGER2 promoter methylation, and the fibrotic fibroblasts showed increased global DNA methylation. The above studies demonstrate that the fibrotic properties of fibroblasts are highly correlated with DNA methylation, and zebularine may inhibit the proliferation of several cancer cells and fibroblasts via demethylation.

Although zebularine and 5AC are equally potent as small oligodeoxynucleotides, the potency of zebularine is approximately one-tenth of that of 5AC in inducing demethylation when used as a single agent (Cheng et al., 2003). This difference is due to lower incorporation of zebularine into DNA and less efficient metabolic activation. Thus, the metabolite of zebularine is only ribonucleotide (RNA) when reverse-phase chromatography analysis is performed on selected cell extracts, and the concentrations of formed deoxyribonucleotide are low (Ben-Kasus et al., 2005). The complex metabolism of zebularine and its inefficient incorporation into DNA explain its lower efficiency than 5AC or DAC, requiring higher doses to equivalently inhibit DNMT.

#### 4.1.3 Guadecitabine

Guadecitabine (SGI-110), also a second-generation DNA methyltransferase inhibitor, is a dinucleotide molecule linked by a phosphodiester bond between DAC and deoxyguanosine (Roboz et al., 2018). DAC is the active component that is responsible for the DNA demethylation effect. The combined nucleotide structure provides drug clearance protection from deamination by cytidine deaminase, thus producing greater biological stability than DAC (Drummond et al., 2014; Kantarjian et al., 2017). DAC can only be administered intravenously, while guadecitabine can be delivered by both subcutaneous and intraperitoneal injection (Chuang et al., 2010). After subcutaneous injection, guadecitabine slowly releases its active metabolite DAC, with an effective half-life up to 8 h longer than that of intravenous administration (approximately 3∼4 h). Furthermore, subcutaneous injection reduces the maximum plasma concentration, which improves the drug effect and prevents peak-related toxicity (Drummond et al., 2014). Therefore, guadecitabine presents high stability and safety, easy administration, prolonged contact time, and a longer half-life compared with that of DAC (Kantarjian et al., 2017).

Loss of DNMT1 expression results in prolonged activation and promoter DNA demethylation of tumor-testis antigens, including the melanoma-associated antigen (MAGEA)-1 and MAGEA3 genes in human fibroblasts. *In vitro*, Chuang et al. (Chuang et al., 2010) demonstrated that guadecitabine is a potent DNA methylation inhibitor that is not susceptible to deamination by cytidine deaminase and is more stable than DAC in tumor-free mice, thereby producing more durable and stable demethylation to reduce fibrosis. *In vivo*, both guadecitabine and zebularine effectively induce the expression of tumor suppressor gene p16 and reduce DNA methylation in the promoter region of p16, thereby inhibiting cancer progression (Srivastava et al., 2015). Therefore, guadecitabine is a potential alternative to DAC. As DAC is an active metabolite of guadecitabine, it also presents teratogenic effects. *In vitro* studies on human liver microsomes suggest that DAC is unlikely to inhibit or induce cytochrome P450 enzymes (Kantarjian et al., 2017). Therefore, guadecitabine is unlikely to participate in significant drug interactions (Griffiths et al., 2013).

### 4.2 Non-nucleotide analog DNA methylation inhibitors

The use of nucleoside analogs is affected by pharmacokinetics, chemical instability, adverse toxic effects, and lack of selectivity. Methylation inhibitors are small molecules that are not incorporated into the DNA and may eliminate many defects associated with nucleoside analogs (Fandy, 2009). In the past two decades, non-nucleoside methylation inhibitors have emerged. Based on their mechanisms of action, these inhibitors are divided into three categories: DNA binders, S-adenosylmethionine (SAM) antagonists, and a subset of drugs with unknown mechanisms.

Procainamide and hydralazine are FDA-approved drugs for cardiovascular disease therapy and exhibit DNA demethylation activity in cloned T cell lines (Cheng et al., 2019). Procainamide downregulates hypermethylation of the anti-inflammatory gene IL27RA in the lung, ear, nose, and throat tissues by inhibiting DNMT1 and 5 mC, thus treating multiple organ failure caused by endotoxin (Shih et al., 2016). Procainamide also reverses hypermethylation of pi-class glutathione S-transferase P1 in tumor tissues and inhibits tumor cell growth (Li et al., 2021). Both hydralazine and procainamide bind to GC-rich regions to induce demethylation, which activates tumor suppressor genes that have been inactivated by hypermethylation to exert anti-tumor activity (Zhao et al., 2012). Although these drugs are chemically stable, present few toxic side effects, and can be administered orally, they require high doses to exert their demethylation effects, which exceed clinically safe doses (Uddin and Fandy, 2021).

RG108 (N-phthalyl-L-tryptophan) is a selective DNMT1 inhibitor and the first small-molecule non-nucleoside DNMT inhibitor to induce demethylation effects in cancer cells (Wu et al., 2019). Using an indole moiety, RG108 binds to the SAM binding site of DNMT1, competitively occupying the SAM site and inhibiting DNA methylation progression. *In vitro* studies in the colon cancer cell line HCT116 reveal that RG108 reactivates the expression of tumor suppressor genes, including p16, TIMP3, and SFRP1, *via* promoter demethylation (Brueckner et al., 2005). Although RG108 does not demonstrate cytotoxicity in HCT116 cells at high concentrations, RG108 activity fails to reverse methylation at the three CpG sites as determined by DNA pyrosequencing within the LINE-1 sequence of leukemia cells compared with the effects of DAC (Carraway et al., 2020). Moreover, high concentrations of RG108 slightly reduce methylation (by 3%–5%). In these experiments, DAC served as a positive control and induced a 25% reduction in methylation at 100-fold lower concentrations than RG108, indicating a large difference in activity between RG108 and DAC(80).

Curcumin, a polyphenolic natural compound derived from *Curcuma longa*, exerts anti-inflammatory, antioxidant, and anti-cancer effects through a variety of epigenetic mechanisms, including DNA methylation, histone modification, and non-coding RNA (Jin et al., 2022). Combining curcumin derivatives and nucleoside DNMT inhibitors may maximize their effects on gene expression, which is more effective than the function of curcumin alone to induce DNA methylation changes *via* the nuclear factor-kappa B, PI3K, Ras, and other signaling pathways (Chen et al., 2007). Curcumin enhances the demethylation effect of DAC on related tumor suppressor genes, upregulates the expression of tumor suppressor genes, and inhibits tumor cell growth (Chen J. Q et al., 2021). Despite extensive research on curcumin and its analogs, no related drugs are currently approved by the FDA for clinical applications. As curcumin is considered a pan-assay interference compound that does not act on the target but shows false positive results, and presents low bioavailability and rapid metabolism, any future clinical relevance of curcumin remains controversial (Uddin and Fandy, 2021).

Lastly, shikonin is a natural naphthoquinone extracted from the shikonin plant, a traditional Chinese medicine, that upregulates the transcriptional activity of tumor suppressor p16 by inhibiting the expression of DMNT1 (Jang et al., 2015; Boulos et al., 2019). Isofitsularin-3, an alkaloid natural product isolated from *Aplysina aerophoba*, moderately inhibits DNMT1) (Florean et al., 2016; Shen and Luo, 2020; Wong, 2021). Moreover, psammaplin, a group of natural compounds derived from the South China Sea sponge *Pseudoceratina purpurea*, inhibits both DNMT and histone deacetylase (HDAC) with low toxicity (Liu et al., 2017). Epigallocatechin gallate, a major component of green tea, non-covalently binds to the catalytic active site of DNMT, thereby inhibiting DNA methylation of related oncogenes (Ni et al., 2011; Yang et al., 2020). As DNMT plays an important role in DNA methylation, DNMT inhibition may block the hypermethylation and downregulate related genes (such as PTGIS, PTEN, and Smad2/Smad3), inhibit the activation and proliferation of HSCs, inhibit the expression of α-SMA and COL1A1 *in vitro*, and delay liver fibrosis.

## 5 Summary and outlook

With enhanced research progress, our understanding of HSC gene regulation and traditional gene regulation mechanisms have been enriched. Activated HSCs are a major cell source of ECM-secreting myofibroblasts, and their activation, proliferation, apoptosis, and aging are key factors in the occurrence and remission of liver fibrosis. The molecular mechanisms underlying HSC activation and liver fibrosis are being translated into successful novel therapeutic approaches. Several studies on DNA methylation-related regulation of HSC biology and function indicate that partial DNA methylation inhibitors upregulate genes that are downregulated in expression in HSCs due to hypermethylation levels and slow the liver fibrosis process. Thus, DNA methylation inhibitors are potential tools for the prevention, diagnosis, treatment, and prognosis of liver fibrosis. Cell-targeted drug therapy for HSCs may emerge as an important method for treating liver fibrosis and as a target for future drug therapy.

## References

[B1] An P. Wei L. L. Zhao S. Sverdlov D. Y. Vaid K. A. Miyamoto M. (2020). Hepatocyte mitochondria-derived danger signals directly activate hepatic stellate cells and drive progression of liver fibrosis. Nat. Commun. 11 (1), 2362. 10.1038/s41467-020-16092-0 32398673 PMC7217909

[B2] Bechtel W. McGoohan S. Zeisberg E. M. Muller G. A. Kalbacher H. Salant D. J. (2010). Methylation determines fibroblast activation and fibrogenesis in the kidney. Nat. Med. 16 (5), 544–550. 10.1038/nm.2135 20418885 PMC3106179

[B3] Ben-Kasus T. Ben-Zvi Z. Marquez V. E. Kelley J. A. Agbaria R. (2005). Metabolic activation of zebularine, a novel DNA methylation inhibitor, in human bladder carcinoma cells. Biochem. Pharmacol. 70 (1), 121–133. 10.1016/j.bcp.2005.04.010 15885659

[B4] Bian E. B. Huang C. Wang H. Chen X. X. Zhang L. Lv X. W. (2014). Repression of Smad7 mediated by DNMT1 determines hepatic stellate cell activation and liver fibrosis in rats. Toxicol. Lett. 224 (2), 175–185. 10.1016/j.toxlet.2013.10.038 24211420

[B5] Boulos J. C. Rahama M. Hegazy M. F. Efferth T. (2019). Shikonin derivatives for cancer prevention and therapy. Cancer Lett. 459, 248–267. 10.1016/j.canlet.2019.04.033 31132429

[B6] Bragelmann J. Barahona Ponce C. Marcelain K. Roessler S. Goeppert B. Gallegos I. (2021). Epigenome-wide analysis of methylation changes in the sequence of gallstone disease, dysplasia, and gallbladder cancer. Hepatology 73 (6), 2293–2310. 10.1002/hep.31585 33020926

[B7] Brueckner B. Garcia Boy R. Siedlecki P. Musch T. Kliem H. C. Zielenkiewicz P. (2005). Epigenetic reactivation of tumor suppressor genes by a novel small-molecule inhibitor of human DNA methyltransferases. Cancer Res. 65 (14), 6305–6311. 10.1158/0008-5472.CAN-04-2957 16024632

[B8] Call J. A. Voelker K. A. Wolff A. V. McMillan R. P. Evans N. P. Hulver M. W. (2008). Endurance capacity in maturing mdx mice is markedly enhanced by combined voluntary wheel running and green tea extract. J. Appl. Physiol. (1985) 105 (3), 923–932. 10.1152/japplphysiol.00028.2008 18583385 PMC2536821

[B9] Carraway H. E. Malkaram S. A. Cen Y. Shatnawi A. Fan J. Ali H. E. A. (2020). Activation of SIRT6 by DNA hypomethylating agents and clinical consequences on combination therapy in leukemia. Sci. Rep. 10 (1), 10325. 10.1038/s41598-020-67170-8 32587297 PMC7316973

[B10] Cebola I. Custodio J. Munoz M. Diez-Villanueva A. Pare L. Prieto P. (2015). Epigenetics override pro-inflammatory PTGS transcriptomic signature towards selective hyperactivation of PGE2 in colorectal cancer. Clin. Epigenetics 7 (1), 74. 10.1186/s13148-015-0110-4 26207152 PMC4512023

[B11] Chen D. Xie P. Cao J. W. (2019). Bioinformatics analysis of the expression and mechanisms of SPP1 gene in hepatocellular carcinoma. Med. J. Wuhan Univ. 40 (6), 886–890.

[B12] Chen K. Zhang Y. Qian L. Wang P. (2021). Emerging strategies to target RAS signaling in human cancer therapy. J. Hematol. Oncol. 14 (1), 116. 10.1186/s13045-021-01127-w 34301278 PMC8299671

[B13] Chen J. Q. Qu C. S. Zhu H. J. Wang W. Zhang X. Y. (2021). Effect of curcumin on RASSF2A expression and promoter methylation in multiple myeloma RPMI-8226 cells. Chin. J. Health Lab. Tec. 31 (23), 2887–2889+93.

[B14] Chen X. Li W. X. Chen Y. Li X. F. Li H. D. Huang H. M. (2018). Suppression of SUN2 by DNA methylation is associated with HSCs activation and hepatic fibrosis. Cell. Death Dis. 9 (10), 1021. 10.1038/s41419-018-1032-9 30282980 PMC6170444

[B15] Chen Y. Choi S. S. Michelotti G. A. Chan I. S. Swiderska-Syn M. Karaca G. F. (2012). Hedgehog controls hepatic stellate cell fate by regulating metabolism. Gastroenterology 143 (5), 1319–1329. e11. 10.1053/j.gastro.2012.07.115 22885334 PMC3480563

[B16] Chen Y. Shu W. Chen W. Wu Q. Liu H. Cui G. (2007). Curcumin, both histone deacetylase and p300/CBP-specific inhibitor, represses the activity of nuclear factor kappa B and Notch 1 in Raji cells. Basic Clin. Pharmacol. Toxicol. 101 (6), 427–433. 10.1111/j.1742-7843.2007.00142.x 17927689

[B17] Chen Z. Zhang Y. (2020). Role of mammalian DNA methyltransferases in development. Annu. Rev. Biochem. 89, 135–158. 10.1146/annurev-biochem-103019-102815 31815535

[B18] Cheng D. Chai J. Wang H. Fu L. Peng S. Ni X. (2021). Hepatic macrophages: Key players in the development and progression of liver fibrosis. Liver Int. 41 (10), 2279–2294. 10.1111/liv.14940 33966318

[B19] Cheng J. C. Matsen C. B. Gonzales F. A. Ye W. Greer S. Marquez V. E. (2003). Inhibition of DNA methylation and reactivation of silenced genes by zebularine. J. Natl. Cancer Inst. 95 (5), 399–409. 10.1093/jnci/95.5.399 12618505

[B20] Cheng Y. He C. Wang M. Ma X. Mo F. Yang S. (2019). Targeting epigenetic regulators for cancer therapy: Mechanisms and advances in clinical trials. Signal Transduct. Target Ther. 4, 62. 10.1038/s41392-019-0095-0 31871779 PMC6915746

[B21] Chitu V. Nacu V. Charles J. F. Henne W. M. McMahon H. T. Nandi S. (2012). PSTPIP2 deficiency in mice causes osteopenia and increased differentiation of multipotent myeloid precursors into osteoclasts. Blood 120 (15), 3126–3135. 10.1182/blood-2012-04-425595 22923495 PMC3471520

[B22] Chuang J. C. Warner S. L. Vollmer D. Vankayalapati H. Redkar S. Bearss D. J. (2010). S110, a 5-Aza-2'-deoxycytidine-containing dinucleotide, is an effective DNA methylation inhibitor *in vivo* and can reduce tumor growth. Mol. Cancer Ther. 9 (5), 1443–1450. 10.1158/1535-7163.MCT-09-1048 20442312 PMC2868087

[B23] Cohen-Naftaly M. Friedman S. L. (2011). Current status of novel antifibrotic therapies in patients with chronic liver disease. Ther. Adv. Gastroenterol. 4 (6), 391–417. 10.1177/1756283X11413002 PMC3187682 22043231

[B24] Daher-Reyes G. S. Merchan B. M. Yee K. W. L. (2019). Guadecitabine (SGI-110): An investigational drug for the treatment of myelodysplastic syndrome and acute myeloid leukemia. Expert Opin. Investig. Drugs 28 (10), 835–849. 10.1080/13543784.2019.1667331 31510809

[B25] De Luca A. Pierno S. Liantonio A. Cetrone M. Camerino C. Fraysse B. (2003). Enhanced dystrophic progression in mdx mice by exercise and beneficial effects of taurine and insulin-like growth factor-1. J. Pharmacol. Exp. Ther. 304 (1), 453–463. 10.1124/jpet.102.041343 12490622

[B26] Ding Y. M. Yu Z. F. Liao X. F. (2015). Effects of Zebularine on cell apoptosis in hepatocarcinoma HepG2 cells and its mechanism. Chin. J. Gastroenterol. Hepatol. 24 (05), 515–518.

[B27] Dooley S. Hamzavi J. Breitkopf K. Wiercinska E. Said H. M. Lorenzen J. (2003). Smad7 prevents activation of hepatic stellate cells and liver fibrosis in rats. Gastroenterology 125 (1), 178–191. 10.1016/s0016-5085(03)00666-8 12851882

[B28] Dooley S. Hamzavi J. Ciuclan L. Godoy P. Ilkavets I. Ehnert S. (2008). Hepatocyte-specific Smad7 expression attenuates TGF-beta-mediated fibrogenesis and protects against liver damage. Gastroenterology 135 (2), 642–659. 10.1053/j.gastro.2008.04.038 18602923

[B29] Drummond M. W. Pocock C. Boissinot M. Mills J. Brown J. Cauchy P. (2014). A multi-centre phase 2 study of azacitidine in chronic myelomonocytic leukaemia. Leukemia 28 (7), 1570–1572. 10.1038/leu.2014.85 24569776

[B30] Duncan E. J. Gluckman P. D. Dearden P. K. (2014). Epigenetics, plasticity, and evolution: How do we link epigenetic change to phenotype? J. Exp. Zool. B Mol. Dev. Evol. 322 (4), 208–220. 10.1002/jez.b.22571 24719220

[B31] Fandy T. E. (2009). Development of DNA methyltransferase inhibitors for the treatment of neoplastic diseases. Curr. Med. Chem. 16 (17), 2075–2085. 10.2174/092986709788612738 19519382

[B32] Florean C. Schnekenburger M. Lee J. Y. Kim K. R. Mazumder A. Song S. (2016). Discovery and characterization of Isofistularin-3, a marine brominated alkaloid, as a new DNA demethylating agent inducing cell cycle arrest and sensitization to TRAIL in cancer cells. Oncotarget 7 (17), 24027–24049. 10.18632/oncotarget.8210 27006469 PMC5029682

[B33] Friedman S. L. (2008). Mechanisms of hepatic fibrogenesis. Gastroenterology 134 (6), 1655–1669. 10.1053/j.gastro.2008.03.003 18471545 PMC2888539

[B34] Gao J. Wei B. de Assuncao T. M. Liu Z. Hu X. Ibrahim S. (2020). Hepatic stellate cell autophagy inhibits extracellular vesicle release to attenuate liver fibrosis. J. Hepatol. 73 (5), 1144–1154. 10.1016/j.jhep.2020.04.044 32389810 PMC7572579

[B35] Griffiths E. A. Choy G. Redkar S. Taverna P. Azab M. Karpf A. R. (2013). SGI-110: DNA methyltransferase inhibitor oncolytic. Drugs Future 38 (8), 535–543. 10.1358/dof.2013.038.08.1980499 26190889 PMC4503259

[B36] Guan H. Zhu N. Tang G. Du Y. Wang L. Yuan W. (2022). DNA methyltransferase 1 knockdown reverses PTEN and VDR by mediating demethylation of promoter and protects against renal injuries in Hepatitis B virus-associated glomerulonephritis. Cell. Biosci. 12 (1), 98. 10.1186/s13578-022-00835-1 35765066 PMC9238139

[B37] Han M. Li J. Cao Y. Huang Y. Li W. Zhu H. (2020). A role for LSH in facilitating DNA methylation by DNMT1 through enhancing UHRF1 chromatin association. Nucleic Acids Res. 48 (21), 12116–12134. 10.1093/nar/gkaa1003 33170271 PMC7708066

[B38] Han P. Hou Y. Zhao Y. Liu Y. Yu T. Sun Y. (2021). Low-dose decitabine modulates T-cell homeostasis and restores immune tolerance in immune thrombocytopenia. Blood 138 (8), 674–688. 10.1182/blood.2020008477 33876188 PMC8394906

[B39] Heinemeier K. M. Skovgaard D. Bayer M. L. Qvortrup K. Kjaer A. Kjaer M. (2012). Uphill running improves rat Achilles tendon tissue mechanical properties and alters gene expression without inducing pathological changes. J. Appl. Physiol. (1985) 113 (5), 827–836. 10.1152/japplphysiol.00401.2012 22797314

[B40] Hsieh T. H. Chien C. L. Lee Y. H. Lin C. I. Hsieh J. Y. Chao M. E. (2014). Downregulation of SUN2, a novel tumor suppressor, mediates miR-221/222-induced malignancy in central nervous system embryonal tumors. Carcinogenesis 35 (10), 2164–2174. 10.1093/carcin/bgu105 24832085

[B41] Huang S. K. Fisher A. S. Scruggs A. M. White E. S. Hogaboam C. M. Richardson B. C. (2010). Hypermethylation of PTGER2 confers prostaglandin E2 resistance in fibrotic fibroblasts from humans and mice. Am. J. Pathol. 177 (5), 2245–2255. 10.2353/ajpath.2010.100446 20889571 PMC2966784

[B42] Huang Y. H. Kuo H. C. Yang Y. L. Wang F. S. (2019). MicroRNA-29a is a key regulon that regulates BRD4 and mitigates liver fibrosis in mice by inhibiting hepatic stellate cell activation. Int. J. Med. Sci. 16 (2), 212–220. 10.7150/ijms.29930 30745801 PMC6367521

[B43] Ivanov A. I. Le H. T. Naydenov N. G. Rieder F. (2021). Novel functions of the septin cytoskeleton: Shaping up tissue inflammation and fibrosis. Am. J. Pathol. 191 (1), 40–51. 10.1016/j.ajpath.2020.09.007 33039354 PMC7786077

[B44] Jang S. Y. Hong D. Jeong S. Y. Kim J. H. (2015). Shikonin causes apoptosis by up-regulating p73 and down-regulating ICBP90 in human cancer cells. Biochem. Biophys. Res. Commun. 465 (1), 71–76. 10.1016/j.bbrc.2015.07.131 26235879

[B45] Jin H. Wang X. Ying J. Wong A. H. Cui Y. Srivastava G. (2007). Epigenetic silencing of a Ca(2+)-regulated Ras GTPase-activating protein RASAL defines a new mechanism of Ras activation in human cancers. Proc. Natl. Acad. Sci. U. S. A. 104 (30), 12353–12358. 10.1073/pnas.0700153104 17640920 PMC1941473

[B46] Jin N. Tu S. J. Zhou Y. (2022). Epigenetic progress of curcumin against cancer. Pharm. Clin. Chin. Materia Medica 13 (03), 113–123.

[B47] Kantarjian H. M. Roboz G. J. Kropf P. L. Yee K. W. L. O'Connell C. L. Tibes R. (2017). Guadecitabine (SGI-110) in treatment-naive patients with acute myeloid leukaemia: Phase 2 results from a multicentre, randomised, phase 1/2 trial. Lancet Oncol. 18 (10), 1317–1326. 10.1016/S1470-2045(17)30576-4 28844816 PMC5925750

[B48] Khatun M. Ray R. B. (2019). Mechanisms underlying hepatitis C virus-associated hepatic fibrosis. Cells 8 (10), 1249. 10.3390/cells8101249 31615075 PMC6829586

[B49] Kolfschoten I. G. van Leeuwen B. Berns K. Mullenders J. Beijersbergen R. L. Bernards R. (2005). A genetic screen identifies PITX1 as a suppressor of RAS activity and tumorigenicity. Cell. 121 (6), 849–858. 10.1016/j.cell.2005.04.017 15960973

[B50] Komatsu Y. Waku T. Iwasaki N. Ono W. Yamaguchi C. Yanagisawa J. (2012). Global analysis of DNA methylation in early-stage liver fibrosis. BMC Med. Genomics 5, 5. 10.1186/1755-8794-5-5 22281153 PMC3295686

[B51] Kong D. Zhang Z. Chen L. Huang W. Zhang F. Wang L. (2020). Curcumin blunts epithelial-mesenchymal transition of hepatocytes to alleviate hepatic fibrosis through regulating oxidative stress and autophagy. Redox Biol. 36, 101600. 10.1016/j.redox.2020.101600 32526690 PMC7287144

[B52] Kong D. S. Zhang F. Qiu P. Zheng S. Z. (2014). Role and mechanisms of DNA methylation in liver diseases. World Chin. J. Dig. 22 (21), 3041–3047. 10.11569/wcjd.v22.i21.3041

[B53] Kovacic J. C. Mercader N. Torres M. Boehm M. Fuster V. (2012). Epithelial-to-mesenchymal and endothelial-to-mesenchymal transition: From cardiovascular development to disease. Circulation 125 (14), 1795–1808. 10.1161/CIRCULATIONAHA.111.040352 22492947 PMC3333843

[B54] Lao V. V. Darwanto A. Sowers L. C. (2010). Impact of base analogues within a CpG dinucleotide on the binding of DNA by the methyl-binding domain of MeCP2 and methylation by DNMT1. Biochemistry 49 (47), 10228–10236. 10.1021/bi1011942 20979427 PMC2996885

[B55] Lei K. Zhu X. Xu R. Shao C. Xu T. Zhuang Y. (2012). Inner nuclear envelope proteins SUN1 and SUN2 play a prominent role in the DNA damage response. Curr. Biol. 22 (17), 1609–1615. 10.1016/j.cub.2012.06.043 22863315 PMC3466333

[B56] Li B. Ma Y. Li Y. Cheng Z. X. (2021). Methylation conditions of SOCS1, GSTP1 and DKK3 genes in liver cancer tissues and their relationships with clinical pathology. Med Pharm J Chin PLA. 33 (10), 37–40+53.

[B57] Li N. Zhu C. C. Xiao R. H. Zhang W. L. Liu Y. M. (2013). Effects of phosphatidyl inositol 3-kinase inhibitors on proliferation and type Ⅰ collagen expression of hepatic stellate cell stimulated by carbon tetrachloride *in vitro* . J. Xi'an Jiaot. Univ. Sci. 34 (2), 164–167.

[B58] Li W. Ma X. Wang F. Chen S. Guo Q. Sun F. (2022). SNHG3 affects gastric cancer development by regulating SEPT9 methylation. J. Oncol. 2022, 3433406. 10.1155/2022/3433406 35528235 PMC9071877

[B59] Liang Y. Yang X. Ma L. Cai X. Wang L. Yang C. (2013). Homocysteine-mediated cholesterol efflux via ABCA1 and ACAT1 DNA methylation in THP-1 monocyte-derived foam cells. Acta Biochim. Biophys. Sin. (Shanghai). 45 (3), 220–228. 10.1093/abbs/gms119 23305686

[B60] Liang S. Cuevas G. Tizani S. Salas T. Liu H. Li B. (2013). Novel mechanism of regulation of fibrosis in kidney tumor with tuberous sclerosis. Mol. Cancer 12, 49. 10.1186/1476-4598-12-49 23705901 PMC3681649

[B61] Liu H. H. Meng Q. K. Shi G. (2017). Effect of Psammaplin A on proliferation and apoptosis of colon cancer SW480 cells *in vitro* . Shandong Med. J. 57 (09), 5–8.

[B62] Mann J. Chu D. C. Maxwell A. Oakley F. Zhu N. L. Tsukamoto H. (2010). MeCP2 controls an epigenetic pathway that promotes myofibroblast transdifferentiation and fibrosis. Gastroenterology 138 (2), 705–714. 10.1053/j.gastro.2009.10.002 19843474 PMC2819585

[B63] Marquez V. E. Eritja R. Kelley J. A. Vanbemmel D. Christman J. K. (2003). Potent inhibition of HhaI DNA methylase by the aglycon of 2-(1H)-pyrimidinone riboside (zebularine) at the GCGC recognition domain. Ann. N. Y. Acad. Sci. 1002, 154–164. 10.1196/annals.1281.014 14751833

[B64] Masszi A. Kapus A. (2011). Smaddening complexity: The role of Smad3 in epithelial-myofibroblast transition. Cells Tissues Organs 193 (1-2), 41–52. 10.1159/000320180 21051861

[B65] Mellen M. Ayata P. Dewell S. Kriaucionis S. Heintz N. (2012). MeCP2 binds to 5hmC enriched within active genes and accessible chromatin in the nervous system. Cell. 151 (7), 1417–1430. 10.1016/j.cell.2012.11.022 23260135 PMC3653293

[B66] Mou Y. Wu G. R. Wang Q. Pan T. Zhang L. Xu Y. (2022). Macrophage-targeted delivery of siRNA to silence Mecp2 gene expression attenuates pulmonary fibrosis. Bioeng. Transl. Med. 7 (2), e10280. 10.1002/btm2.10280 35600643 PMC9115697

[B67] Nakamura K. Aizawa K. Nakabayashi K. Kato N. Yamauchi J. Hata K. (2013). DNA methyltransferase inhibitor zebularine inhibits human hepatic carcinoma cells proliferation and induces apoptosis. PLoS One 8 (1), e54036. 10.1371/journal.pone.0054036 23320119 PMC3540068

[B68] Ni J. Xu W. J. Wang X. (2011). The epigenetic mechanism of tea polyphenols in tumor prevention. Int. J. Genet. (02), 107–112.

[B69] Ooi S. K. Qiu C. Bernstein E. Li K. Jia D. Yang Z. (2007). DNMT3L connects unmethylated lysine 4 of histone H3 to de novo methylation of DNA. Nature 448 (7154), 714–717. 10.1038/nature05987 17687327 PMC2650820

[B70] Pan X. Y. Yang Y. Meng H. W. Li H. D. Chen X. Huang H. M. (2018). DNA methylation of PTGIS enhances hepatic stellate cells activation and liver fibrogenesis. Front. Pharmacol. 9, 553. 10.3389/fphar.2018.00553 29892223 PMC5985735

[B71] Pellicoro A. Ramachandran P. Iredale J. P. Fallowfield J. A. (2014). Liver fibrosis and repair: Immune regulation of wound healing in a solid organ. Nat. Rev. Immunol. 14 (3), 181–194. 10.1038/nri3623 24566915

[B72] Peng D. Fu M. Wang M. Wei Y. Wei X. (2022). Targeting TGF-beta signal transduction for fibrosis and cancer therapy. Mol. Cancer 21 (1), 104. 10.1186/s12943-022-01569-x 35461253 PMC9033932

[B73] Perugorria M. J. Wilson C. L. Zeybel M. Walsh M. Amin S. Robinson S. (2012). Histone methyltransferase ASH1 orchestrates fibrogenic gene transcription during myofibroblast transdifferentiation. Hepatology 56 (3), 1129–1139. 10.1002/hep.25754 22488473 PMC3430805

[B74] Ren J. J. Huang T. J. Zhang Q. Q. Zhang H. Y. Guo X. H. Fan H. Q. (2019). Insulin-like growth factor binding protein related protein 1 knockdown attenuates hepatic fibrosis via the regulation of MMPs/TIMPs in mice. Hepatobiliary Pancreat. Dis. Int. 18 (1), 38–47. 10.1016/j.hbpd.2018.08.008 30243878

[B75] Roboz G. J. Kantarjian H. M. Yee K. W. L. Kropf P. L. O'Connell C. L. Griffiths E. A. (2018). Dose, schedule, safety, and efficacy of guadecitabine in relapsed or refractory acute myeloid leukemia. Cancer 124 (2), 325–334. 10.1002/cncr.31138 29211308 PMC5814873

[B76] Shen H. Laird P. W. (2013). Interplay between the cancer genome and epigenome. Cell. 153 (1), 38–55. 10.1016/j.cell.2013.03.008 23540689 PMC3648790

[B77] Shen Q. Y. Luo W. S. (2020). Research progress of epigenetic mechanism and hepatic fibrosis. Guangxi Med. J. 42 (6), 755–758.

[B78] Shi M. M. Tang C. Xin Y. (2022). 5-Azacytidine regulates RNA methylation in COVID-19 virus. Int. J. Geriatrics 43 (3), 263–267.

[B79] Shih C. C. Liao M. H. Hsiao T. S. Hii H. P. Shen C. H. Chen S. J. (2016). Procainamide inhibits DNA methylation and alleviates multiple organ dysfunction in rats with endotoxic shock. PLoS One 11 (9), e0163690. 10.1371/journal.pone.0163690 27661616 PMC5035080

[B80] Song H. Chen L. Liu W. Xu X. Zhou Y. Zhu J. (2021). Depleting long noncoding RNA HOTAIR attenuates chronic myelocytic leukemia progression by binding to DNA methyltransferase 1 and inhibiting PTEN gene promoter methylation. Cell. Death Dis. 12 (5), 440. 10.1038/s41419-021-03637-4 33941772 PMC8093289

[B81] Song Z. Chen W. Athavale D. Ge X. Desert R. Das S. (2021). Osteopontin takes center stage in chronic liver disease. Hepatology 73 (4), 1594–1608. 10.1002/hep.31582 32986864 PMC8106357

[B82] Srivastava P. Paluch B. E. Matsuzaki J. James S. R. Collamat-Lai G. Taverna P. (2015). Immunomodulatory action of the DNA methyltransferase inhibitor SGI-110 in epithelial ovarian cancer cells and xenografts Epigenetics 10 (3), 237–246. 10.1080/15592294.2015.1017198 25793777 PMC4623048

[B83] Stemberger C. Matusan-Ilijas K. Avirovic M. Bulat-Kardum L. Ivancic A. Jonjic N. (2014). Osteopontin is associated with decreased apoptosis and αv integrin expression in lung adenocarcinoma. Acta histochem. 116 (1), 222–229. 10.1016/j.acthis.2013.07.009 23992637

[B84] Sun W. Bunn P. Jin C. Little P. Zhabotynsky V. Perou C. M. (2018). The association between copy number aberration, DNA methylation and gene expression in tumor samples. Nucleic Acids Res. 46 (6), 3009–3018. 10.1093/nar/gky131 29529299 PMC5887505

[B85] Tahiliani M. Koh K. P. Shen Y. Pastor W. A. Bandukwala H. Brudno Y. (2009). Conversion of 5-methylcytosine to 5-hydroxymethylcytosine in mammalian DNA by MLL partner TET1. Science 324 (5929), 930–935. 10.1126/science.1170116 19372391 PMC2715015

[B86] Tang M. Lozano Hernandez L. Reginald-Opara J. N. Svirskis D. Leung E. Wang H. (2021). Zebularine suppressed gemcitabine-induced senescence and improved the cellular and plasma pharmacokinetics of gemcitabine, augmented by liposomal co-delivery. Int. J. Pharm. 602, 120659. 10.1016/j.ijpharm.2021.120659 33933647

[B87] Tang X. D. Liu F. (2014). New progress of myelodysplastic syndromes in the 55th ASH annual meeting. J. Leukemia Lymphoma 23 (1), 20–29. 10.3760/cma.j.issn.1009-9921.2014.01.008

[B88] Tao H. Huang C. Yang J. J. Ma T. T. Bian E. B. Zhang L. (2011). MeCP2 controls the expression of RASAL1 in the hepatic fibrosis in rats. Toxicology 290 (2-3), 327–333. 10.1016/j.tox.2011.10.011 22056649

[B89] Tsuchida T. Friedman S. L. (2017). Mechanisms of hepatic stellate cell activation. Nat. Rev. Gastroenterol. Hepatol. 14 (7), 397–411. 10.1038/nrgastro.2017.38 28487545

[B90] Turgay Y. Ungricht R. Rothballer A. Kiss A. Csucs G. Horvath P. (2010). A classical NLS and the SUN domain contribute to the targeting of SUN2 to the inner nuclear membrane. EMBO J. 29 (14), 2262–2275. 10.1038/emboj.2010.119 20551905 PMC2910269

[B91] Uddin M. G. Fandy T. E. (2021). DNA methylation inhibitors: Retrospective and perspective view. Adv. Cancer Res. 152, 205–223. 10.1016/bs.acr.2021.03.007 34353438 PMC10275377

[B92] Wong K. K. (2021). DNMT1: A key drug target in triple-negative breast cancer. Semin. Cancer Biol. 72, 198–213. 10.1016/j.semcancer.2020.05.010 32461152

[B93] Wu C. zhang R. Z. Wang T. S. Zhou X. B. Ya C. Y. Qin X. R. (2021). Role of cytokines in the progression of hepatic fibrosis:A review. J. Hainan Med. Univ. 27 (13), 1036–1040.

[B94] Wu C. F. Zhang D. F. Zhang S. Sun L. Liu Y. Dai J. J. (2019). Optimizing treatment of DNA methyltransferase inhibitor RG108 on porcine fibroblasts for somatic cell nuclear transfer. Reprod. Domest. Anim. 54 (12), 1604–1611. 10.1111/rda.13569 31549747

[B95] Wu Y. Bu F. Yu H. Li W. Huang C. Meng X. (2017). Methylation of Septin9 mediated by DNMT3a enhances hepatic stellate cells activation and liver fibrogenesis. Toxicol. Appl. Pharmacol. 315, 35–49. 10.1016/j.taap.2016.12.002 27939986

[B96] Xie B. T. Li W. Zhang Y. G. (2022). Application value of combined detection of CEA, HSP60 and SEPT9 in early diagnosis and prognosis of color-ectal cancer. Med. J. Chin. People's Liberation Army 34 (6), 17–20.

[B97] Xu F. Liu C. Zhou D. Zhang L. (2016). TGF-β/SMAD pathway and its regulation in hepatic fibrosis. J. Histochem Cytochem 64 (3), 157–167. 10.1369/0022155415627681 26747705 PMC4810800

[B98] Xu J. Liu X. Koyama Y. Wang P. Lan T. Kim I. G. (2014). The types of hepatic myofibroblasts contributing to liver fibrosis of different etiologies. Front. Pharmacol. 5, 167. 10.3389/fphar.2014.00167 25100997 PMC4105921

[B99] Xu J. J. Zhu L. Li H. D. Du X. S. Li J. J. Yin N. N. (2022). DNMT3a-mediated methylation of PSTPIP2 enhances inflammation in alcohol-induced liver injury via regulating STAT1 and NF-κB pathway. Pharmacol. Res. 177, 106125. 10.1016/j.phrs.2022.106125 35149186

[B100] Yang Y. Wu X. Q. Li W. X. Huang H. M. Li H. D. Pan X. Y. (2018). PSTPIP2 connects DNA methylation to macrophage polarization in CCL4-induced mouse model of hepatic fibrosis. Oncogene 37 (47), 6119–6135. 10.1038/s41388-018-0383-0 29993036

[B101] Yang C. Y. Li G. C. Song D. Sun Y. L. (2018). DNA methylation/demethylation-targeted drugs: Challenge and opportunity. Chin. Bull. Life Sci. 30 (04), 383–390.

[B102] Yang J. J. Tao H. Huang C. Shi K. H. Ma T. T. Bian E. B. (2013). DNA methylation and MeCP2 regulation of PTCH1 expression during rats hepatic fibrosis. Cell. Signal 25 (5), 1202–1211. 10.1016/j.cellsig.2013.01.005 23333245

[B103] Yang J. J. Wang J. Yang Y. Yang Y. Li J. Lu D. (2022). ALKBH5 ameliorated liver fibrosis and suppressed HSCs activation via triggering PTCH1 activation in an m(6)A dependent manner. Eur. J. Pharmacol. 922, 174900. 10.1016/j.ejphar.2022.174900 35318034

[B104] Yang L. Zhang W. Chopra S. Kaur D. Wang H. Li M. (2020). The epigenetic modification of epigallocatechin gallate (EGCG) on cancer. Curr. Drug Targets 21 (11), 1099–1104. 10.2174/1389450121666200504080112 32364072

[B105] Yang Z. Xie C. Xu W. Liu G. Cao X. Li W. (2015). Phosphorylation and inactivation of PTEN at residues Ser380/Thr382/383 induced by *Helicobacter pylori* promotes gastric epithelial cell survival through PI3K/Akt pathway. Oncotarget 6 (31), 31916–31926. 10.18632/oncotarget.5577 26376616 PMC4741650

[B106] Yao Q. Y. Xu B. L. Wang J. Y. Liu H. C. Zhang S. C. Tu C. T. (2012). Inhibition by curcumin of multiple sites of the transforming growth factor-beta1 signalling pathway ameliorates the progression of liver fibrosis induced by carbon tetrachloride in rats. BMC Complement. Altern. Med. 12, 156. 10.1186/1472-6882-12-156 22978413 PMC3495222

[B107] Yu F. Chen B. Fan X. Li G. Dong P. Zheng J. (2017). Epigenetically-regulated MicroRNA-9-5p suppresses the activation of hepatic stellate cells via TGFBR1 and TGFBR2. Cell. Physiol. Biochem. 43 (6), 2242–2252. 10.1159/000484303 29073595

[B108] Zhang F. X. Zhang J. D. Shan B. E. Chu J. X. (2021). Zebularine induces apoptosis of esophageal cancer cells via demethylation SFRP2/Dkk3 to regulate Wnt/β-catenin signaling pathway. Acta Pharm. Sin. 56 (05), 1384–1390.

[B109] Zhang T. Lan T. Wei L. Feng T. Huang X. L. (2019). Advances in research on role of methylation and its mechanism in liver fibrosis. Chin. J. Bases Clin. General Surg. 26 (2), 229–235.

[B110] Zhang Z. Chen X. X. Dong R. K. Dong R. F. (2015). The effect of zebularine on p16 mRNA expression in SGC-7901 cell line[J]. Chin. J. Gen. Surg. 24 (06), 889–891.

[B111] Zhao J. Chen J. (2019). Research progress of signaling pathways in liver fibrosis. Chin. J. Hepatology 27 (6), 403–406. 10.3760/cma.j.issn.1007-3418.2019.06.002 PMC12770161 31357752

[B112] Zhao J. Xia Y. B. Zhang Y. S. Zhao G. H. (2012). Hydralazine reverses human gastric cancer cell lines p16 methylation and reactivates its expression. Chin. J. Clin. Pharmacol. Ther. 17 (01), 5–9.

[B113] Zhao L. Y. Li M. R. Liu Y. D. (2022). Detection of SEPT9 gene methylation in peripheral blood in the diagnosis of gastric cancer progress. J. Tianjin Med. Univ. 28 (2), 218–221.

[B114] Zhou P. Lu Y. Sun X. H. (2011). Zebularine suppresses TGF-beta-induced lens epithelial cell-myofibroblast transdifferentiation by inhibiting MeCP2. Mol. Vis. 17, 2717–2723. 22065925 PMC3209433

[B115] Zhu D. D. Su M. T. (2021). Value of FGA、HSP90α、SPP1 combined in the early diagnosis of HBV-associated hepatocellular carcinoma. Pract. J. Cancer 36 (04), 632–635.

[B116] Zhu H. He C. Zhao H. Jiang W. Xu S. Li J. (2020). Sennoside A prevents liver fibrosis by binding DNMT1 and suppressing DNMT1-mediated PTEN hypermethylation in HSC activation and proliferation. FASEB J. 34 (11), 14558–14571. 10.1096/fj.202000494RR 32946656

