# Corrigendum: Role and mechanism of DNA methylation and its inhibitors in hepatic fibrosis

**DOI:** 10.3389/fgene.2023.1265506

**Published:** 2023-08-11

**Authors:** Shi-Yi Lyu, Wang Xiao, Guang-Zu Cui, Cheng Yu, Huan Liu, Min Lyu, Qian-Ya Kuang, En-Hua Xiao, Yong-Heng Luo

**Affiliations:** ^1^ Department of Radiology, The Second Xiangya Hospital, Central-South University, Changsha, Hunan, China; ^2^ Department of Gastrointestinal Surgery, The Second Xiangya Hospital, Central-South University, Changsha, Hunan, China; ^3^ XiangYa School of Medicine, Central South University, Changsha, Hunan, China

**Keywords:** hepatic fibrosis, DNA methylation, epigenetics, hepatic stellate cells (HSCs), DNA methylation inhibitors

In the published article, there was an error in the **Funding** statement. The statement neglected to mention the funding supported by the Fundamental Research Funds for the Central Universities of Central South University (2023ZZTS0880):

"This study was supported by the Natural Science Foundation of Hunan Province No.2021JJ30945 (to Y-HL) and Center for Medical Imaging in Hunan Province (2020SK4001)."

The correct Funding statement appears below.

"This study was supported by the Natural Science Foundation of Hunan Province No.2021JJ30945 (to Y-HL), Center for Medical Imaging in Hunan Province (2020SK4001) and the Fundamental Research Funds for the Central Universities of Central South University (2023ZZTS0880)."

The authors apologize for this error and state that this does not change the scientific conclusions of the article in any way. The original article has been updated.

